# A Methodology for the Assessment and Prioritization of Genetic Biocontainment Technologies for Engineered Microbes

**DOI:** 10.1089/apb.2023.0025

**Published:** 2024-06-20

**Authors:** Stephen Payne, Scott Wick, Peter A. Carr, Nicholas J. Guido

**Affiliations:** MIT Lincoln Laboratory, Lexington, Massachusetts, USA.

**Keywords:** biocontainment, synthetic genome, engineered organisms, systems analysis, prioritization

## Abstract

**Introduction:**

Organisms engineered with synthetic genes and genomes have the potential to play critical roles to address important issues in the environment, human health, and manufacturing. Engineered genetic biocontainment technologies are needed to manage the risks of unintended consequences when deploying these biological systems in consultation with the biosafety and biosecurity communities. Metrics measuring genetic biocontainment and a methodology to apply them are required to determine which genetic biocontainment technologies warrant further development for real-world applications. In this study, we develop and apply a systems analysis of the current technical landscape using expert opinion and a metric-based scoring system resulting in a semiquantitative comparative assessment of genetic biocontainment technologies in microorganisms.

**Methods:**

Genetic biocontainment technologies were evaluated according to multiple metrics, falling into two broad classes: feasibility and applicability. Specific genetic biocontainment example scenarios and generalized categories were scored with these metrics. Gap analysis was carried out, indicating particular areas where genetic biocontainment can be improved.

**Results:**

Metric analysis scoring of feasibility and applicability enabled prioritization of genetic biocontainment technologies for real-world applications. Gap analysis showed that technology readiness and containment stability scored low for a number of scenarios and categories, indicating a general need for further development before they can be ready for deployment.

**Conclusion:**

Developing an assessment framework with defined metrics produced a straightforward system for evaluating genetic biocontainment strategies intended for various real-world applications. Use of the methodology also provided insights into existing gaps in genetic biocontainment strategies, and by altering the metrics, can be applied to other biotechnologies.

## Introduction

The engineering of biology is a growing and diverse field with significant economic and national security implications, however there is a lack of analysis methods for prioritizing the selection and development of new synthetic organisms for real-world applications. Biocontainment of these synthetic organisms is a notable example of a burgeoning biotechnology requiring prioritization of specific technologies for a multitude of applications.

Specifically, genetic biocontainment, where an organism has been engineered at the genetic level to be constrained to a particular operating environment, will be instrumental in reducing risks of the use of organisms engineered with synthetic genes and genomes.^1^ Engineered organisms and, more specifically, those with synthetic genomes represent relatively recent advances in biotechnology^2–4^ with a number of potential exciting applications, including nitrogen-fixing bacteria^5–7^ to intelligent probiotics^8–10^ and oil-remediating microbes.^11–13^

As these genomes become more common, so too will concerns that the products of such extreme genetic engineering require careful risk management. Biocontainment of organisms with fully synthetic genomes is of particular concern due to the possibility of orthogonality to natural systems.^14^ A microorganism with a synthetic genome harboring an alternate genetic code can, for instance, be resistant to some natural viruses^15^ and, therefore, gain a competitive advantage over native organisms in some environments, thus requiring strict biocontainment.

Such risk management for biological systems has been of particular importance to the biosafety and biosecurity communities, which have historically been engaged in containment of pathogens. With the advancement of synthetic biology these communities will need to be increasingly active in monitoring and containing genetically engineered organisms deliberately deployed in many possible environments.

The main focus of this study is on microbes (typically BSL-1 or Generally Recognized as Safe [GRAS] organisms) engineered for use in an operational environment, which would benefit from genetic biocontainment methods to avoid contamination and unintended spread. Although genetic biocontainment can also be applied to dangerous pathogens, such cases should be carefully considered, would still require existing standard physical containment methods, and are outside the focus of this study.

Genetic biocontainment will likely have a major role to play in maximizing the benefit of engineered organisms while minimizing risks. However, many of the specific implementations of genetic biocontainment require further development and testing before operational use is possible in many environments. Given the wide diversity of new genetic biocontainment technologies as well as their ability to be used for many unique applications, an assessment methodology is critical not only to establish priorities but also for economic allocation.

It has been well established that strategies are needed to address such biotechnology development for industry, government, and military deployment.^16^ To these ends, once requirements for real-world applications are established, it is necessary to choose which specific technologies should be pursued to meet these requirements.

Previous assessment methodologies have been commonly applied to estimate and to prioritize risk (i.e., for financial instruments, national security, and environmental impact) including in biological systems.^17,18^ Similar assessment can be applied to the relative benefits and merits of biotechnologies where such methodologies are often lacking. This type of assessment is applied in this study to the specific biotechnology of genetic biocontainment in microbes.

There has been significant effort invested in developing genetic biocontainment mechanisms, especially within the past 15 years, resulting in a large number of publications and patents.^1,19,20^ Common methods of genetic biocontainment include the use of microbe-targeting toxins, essential genes, or nutritional requirements. More recently, a newer and potentially very effective genetic biocontainment method involving the engineering of the genetic code and the incorporation of nonstandard amino acids (nsAAs) has been demonstrated.^21–23^

Genetic code engineering is especially enabled through the synthesis of entire genomes^3^ with opportunities for genetic isolation and biocontainment that are still being explored. In this study, a methodology is presented for assessing these different genetic biocontainment technologies for the deployment of engineered microbes.

## Methods

The overall process for developing the genetic biocontainment assessment methodology and carrying out that assessment is shown in (Supplementary Figure S1. Relevant genetic biocontainment technologies and their possible applications were identified through literature and patent searches. Next, metrics were created that can be applied to any genetic biocontainment technology regardless of the application or the specifics of the technology.

Specific scenarios were conceived of and scored to test the metrics and the scoring process for both feasibility and applicability. General categories of engineered genetic biocontainment were created to survey the field overall and determine the advantages and shortcomings of each type of containment. Finally, the categories were scored, and all of the scenarios and categories were normalized and compared to assist with prioritizing the technologies considered.

### Survey of the Literature

The process to find publications relevant to genetic biocontainment began with iterative keyword searches in PubMed, Scopus, and Google Scholar. Then, controlled vocabulary terms were used in MeSH from PubMed. A citation search was conducted in Scopus, including articles that reference, or are referenced by, the publications resulting from the first two steps. At each point, a subset of relevant results was selected. Finally, all of the results were combined, duplicates were removed, and the output was formatted for the evaluators to use in the analysis of the technologies.

The search terms appear in Supplementary Table S1. The results of the literature search can be found in Supplementary Table S2. Patents were found with an in depth search of Google patents, Patent Scout, and Espacenet using the same keywords as those for the literature search. As is the case in any information search, not all relevant information will be found in the public domain, such as industrial trade secrets, for example. However, the exhaustive literature and patent search should cover a significant portion of the field.

### Subject Matter Experts

It is important to consider carefully who is qualified to be subject matter experts (SMEs) when applying the metrics and tools discussed throughout. Ideally, SMEs will have >10 years of experience relevant to molecular biology, bioengineering, synthetic biology, and/or biosafety to critically assess each technology through the metrics outlined. It is also desirable to have multiple SMEs from varying perspectives and backgrounds applying these metrics to reduce bias. The SMEs for this study were the authors. Between them, they have ∼80 years of relevant training and work experience. They have held positions at diverse institutions throughout academia, industry, and government advisement.

### Development of Scenarios

To facilitate the development of genetic biocontainment scenarios, specific types of information were evaluated. These types allowed for the analysis to sample a large parameter space with a modest number of scenarios, minimizing overlap, and ensuring that the most information about each scenario was considered. As listed in Supplementary Table S3, the information types considered for each scenario were release condition, containment technology, controller, and containment details. Examples of genetic biocontainment methods from the literature were used to guide scenario development.

### Creation of Generalized Technology Categories

Although the specific scenarios facilitated the assessment of detailed implementations of a genetic biocontainment technology for particular applications, to describe the range of technologies more broadly, generalized categories of technologies are helpful. Generalized genetic biocontainment categories were created to allow for the assessment of many implementations of the same type of genetic biocontainment technology together as a group. These categories were based on the engineering principles of actuators and controllers as applied to genetic biocontainment and examples of those principles in the literature.

Essential genes, toxin genes, nutrient dependency, and genomic recoding were identified as the categories of actuators that cover the majority of the existing technologies. Three types of controllers were identified as environmental switches, user switches, and intrinsic (or no controller). Please note that this methodology is easily adapted to include new categories of actuators as they are developed. These categories were considered separate from any particular use case to assess the technology in general and not as applied to any one application.

### Developing the Metrics and Scoring System

A methodology was developed to quantitatively assess different types of genetic biocontainment. This consists of metrics for feasibility and applicability of containment technologies that can be scored by evaluators with subject matter expertise. Feasibility metrics refer to the challenges of engineering the organism. The metrics for feasibility are based on the design, build, test, learn cycle of engineering, and represent, in part, the resources and expertise needed to achieve a functional containment system, to explore the readiness of a technology for implementation based on its maturity and risk of failure in development.

The applicability category explores the technology fit for use in the field, and the metrics are based on how well the genetic biocontainment will work when utilized in the intended use case. Applicability metrics describe the overall hypothesized performance of the technology for real-world application and focus on the likelihood of escape of the organisms or their genetic material as well as ease of use. The assessment metrics and score descriptions for each metric are shown in Supplementary Tables S4 and S5.

### Scoring, Normalization, and Comparison

To carry out the analysis of particular genetic biocontainment technologies, evaluators (the authors) researched and assessed the metrics described earlier. Each of the metrics in the feasibility and applicability assessments was scored on a scale of 1–3. A score of 1 indicates that the particular technology being assessed does not perform well in that metric, thus limiting the feasibility or applicability of the genetic biocontainment technology. A score of 2 means that the particular technology performs moderately for that metric, neither hindering nor helping feasibility or applicability of genetic biocontainment. A score of 3 indicates that the particular technology being assessed performs well for that metric, favoring feasibility or applicability of genetic biocontainment.

The evaluators agreed that the applicability assessment metric for “escape rate,” which addressed the main concern of how well a technology can achieve genetic biocontainment, was important enough to be weighted (four times) higher than others. The high importance of this metric is inherent to genetic biocontainment technologies as the escape rate is a fundamental measure of their function and quantitatively indicates how many cells with active genetic biocontainment technology will escape into a given environment.

Reviews and resources focused on genetic biocontainment often highlight the escape rate as an important metric.^19,20,24^ In our analyses, weighting the escape rate metric resulted in assessments that were more consistent with evaluator research. It is also important to note that biological organism escapees are by their very nature capable of self-replication. Thus, one escapee cell is enough to have notable consequences in some cases, a fact that supports the importance of minimizing the escape rate metric below detection if possible, depending on the risk assessment of the organism and the release environment.

In addition to weighting of the metrics, normalization was also applied to both the feasibility and applicability scores. Individual scores for all genetic biocontainment technologies were assessed for feasibility and applicability separately. In some cases, metrics did not apply to a particular genetic biocontainment technology and were not scored. Normalization allowed for the direct comparison of technologies even when the assessments had a different number of metrics scored. The normalization was calculated with Equation (1):

$$\begin{array}{cc} & Normalizedscore=\\ & \frac{\left[\left(totalassignedscore\right)-\left(minimallyachievablescore\right)\right]}{\left[\left(maximallyachievablescore\right)-\left(minimallyachievablescore\right)\right]} \end{array}$$

The total assigned score represented the sum of the metric scores assigned to that genetic biocontainment technology by the evaluators. The minimally achievable score was the value if all applicable metrics were scored with a 1. The maximally achievable score was the value if all applicable metrics were scored with a 3.

Three evaluators scored each scenario assessment, whereas individual evaluators assessed each generalized category. The next step was to compare the technologies with each other considering both feasibility and applicability. Plotting the data for each genetic biocontainment technology on a 2-axis graph (feasibility and applicability) provided a more visual comparison for prioritization as opposed to creating a single combined score.

**Figure 1.**
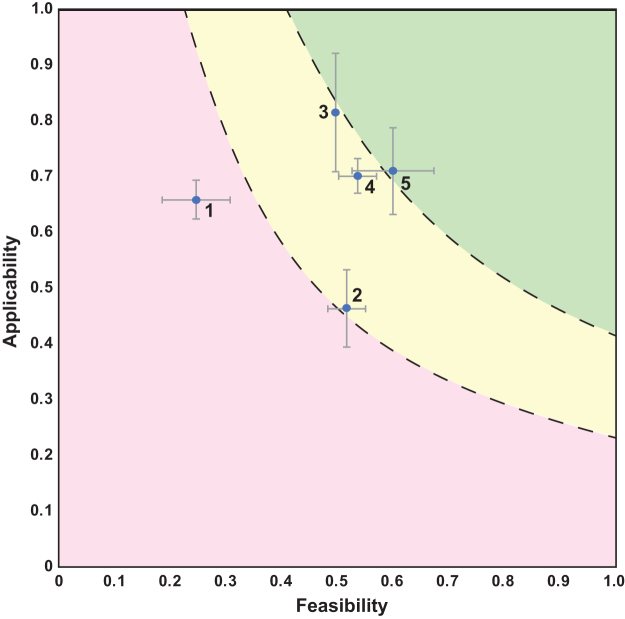
Applicability versus feasibility for genetic biocontainment scenarios. The points represent the average of three assessments carried out by evaluators, whereas the error bars show the standard deviation of both the feasibility and applicability at each point. The numbers next to the data points are associated with those in Table 1. The red region is designated as those technologies that “need basic research development,” yellow represents those that “require optimization,” whereas green represents “trending to advancement/implementation.”

**Figure 2.**
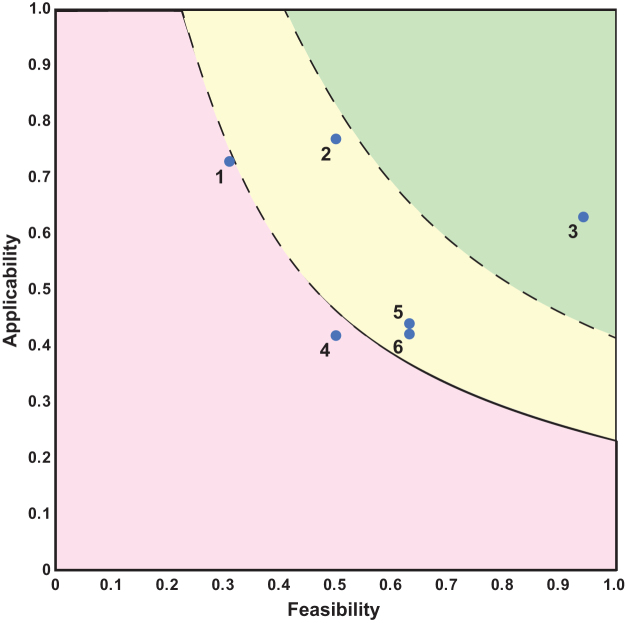
Applicability versus feasibility for genetic biocontainment categories. The numbers next to the data points are associated with those in Table 2. The red, yellow, and green areas are the same percentiles and designations as those in Figure 1.

The 2-axis graphs were divided into three sections representing bins that can help guide prioritization (as seen in Figures 1 and 2). To create the divisions in the graphs, the feasibility and applicability scores were multiplied, creating a combined score for each scenario and category. The 25th percentile and the 75th percentile were found for the set of combined scores, and the equation *y* = *P_i_*/*x* was plotted for each percentile, where *y* is the applicability, *x* is the feasibility, *P* is the calculated percentile number, and *i* is the percentile.

**Table 1.** Applicability and feasibility scores for genetic biocontainment scenarios

***No***.	***Biocontainment scenario***	***Feasibility***	***Weighted applicability***	***Feasibility stdev***	***Applicability stdev***
1	GenCode	0.25	0.65	0.06	0.04
2	Cryodeath	0.52	0.46	0.03	0.07
3	Phosphite	0.50	0.81	0.00	0.11
4	Essential yeast	0.54	0.70	0.03	0.03
5	DAP therapeutic	0.60	0.71	0.08	0.08

Each row is associated with the corresponding data point shown in Figure 1. Average feasibility and applicability are shown along with the stdev of the feasibility and applicability of the three normalized evaluator scores for that scenario.

DAP, diaminopimelate; stdev, standard deviation.

**Table 2.** Applicability and feasibility scores for genetic biocontainment categories

***No***.	***Biocontainment category***	***Feasibility***	***Weighted applicability***
1	Genomic recoding	0.31	0.73
2	Essential gene user switch	0.5	0.77
3	Nutrient dependency	0.94	0.63
4	Essential gene environmental switch	0.5	0.42
5	Toxin gene user switch	0.63	0.44
6	Toxin gene environmental switch	0.63	0.42

Each row is associated with the corresponding data point shown in Figure 2. Average feasibility and applicability are shown.

Below the 25th percentile line (the red section of the 2-axis graphs) was determined to represent those technologies that “need basic research development,” the area between the 25th and 75th percentile (the yellow section) represents those that “require optimization,” whereas the area of the graph above the 75th percentile (green region) represents “trending to advancement/implementation.”

### Gap Analysis

An average of all assessments for each metric in the feasibility and applicability scores across all of the specific scenarios and generalized categories was calculated. The average scores were normalized between 0 and 1 for the feasibility and applicability metric sets separately. A low score for a particular metric across all of the unique technologies evaluated would indicate that there are few solutions successfully fulfilling operational requirements represented by that metric among the scenarios and categories evaluated for this study. This analysis helped to elucidate the potential gaps in the genetic biocontainment state of the art as determined by the evaluator scoring.

## Results

To determine the utility of this genetic biocontainment assessment methodology, various genetic biocontainment technologies were evaluated. To begin testing the methodology, specific examples of genetic biocontainment technologies described in the research literature or used in industry were evaluated to provide more detailed scenarios. The technologies themselves were chosen to represent a diverse set of solutions to genetic biocontainment with varying levels of feasibility and applicability and to serve as concrete examples of genetic biocontainment technologies implemented experimentally.

Importantly, one real-world application scenario, or use case, was envisaged for each of the genetic biocontainment technologies outlined in the literature to assess the viability of such a system in the field. In constructing these scenarios emphasis was placed on sampling a broad range of conditions such that the individual scenarios were unique from one technology to the next.

After these technologies were evaluated using the assessment methodology, additional generalized strategies for real-world genetic biocontainment were also assessed to cover the field more broadly. The overall process for developing the genetic biocontainment assessment methodology and carrying out that assessment is shown in Supplementary Figure S1. Specific categories of information evaluated for scenario development are listed in Supplementary Table S3.

### Application Scenarios

The following scenarios cover a wide range of different release conditions, containment methods, and controllers. Complete assessment scoring using the metrics developed can be found in Supplementary Table S6. The overall normalized prioritization scores are shown in Table 1.

#### Genomic recoding (GenCode)

In the genomic recoding scenario, genetic biocontainment is achieved in a bacterial strain when the genetic code is changed such that nucleotides normally translated as a particular amino acid are reassigned, through genetic alteration, to an nsAA not normally found in nature. That nsAA can be incorporated into essential proteins and would then be required by the cell that will only survive in environments where the nsAA is present (genomic recoding is an involved process described previously^25^).

This approach was demonstrated using an *Escherichia coli* (*E. coli)* strain engineered to only survive in the presence of para-azido-phenylalanine (pAzF).^21^ The scenario constructed for use with the assessment envisioned applying this approach to a nitrogen-fixing *Klebsiella* strain such as that engineered by Pivot Bio to increase corn yields.^5^ In this scenario, crops would be treated with pAzF to ensure growth of the bacteria in the soil of the intended crops but not outside of that environment.

As indicated in Figure 1, the GenCode scenario was scored as moderately applicable but with low feasibility resulting in average assessments of 0.65 and 0.25, respectively. Factors enhancing applicability are the expectation of low cell and genetic material escape rates, and that other organisms would likely have a limited impact on genetic biocontainment. However, the overall applicability score was reduced by the high cost of nsAAs ($252/g for pAzF)^26^ and the potential hazard of the amino acids (the explosiveness of pAzF was of particular concern).^27^

Meanwhile, feasibility was scored low with weaknesses in information availability, technology readiness, expertise required, and engineering complexity, due to the difficulty of engineering such a strain. It is important to realize that that a low score should not be thought of as automatically disqualifying. Rather, a low score may indicate some parts of the solution need further development, or may simply be harder to achieve. As is shown in Figure 1, the scenario falls in the section of the graph delineated as “needs basic research development.”

#### Toxin gene (Cryodeath)

This genetic biocontainment technology scenario consists of *E. coli* containing a genetic circuit with an environmentally sensitive promoter driving transcription of a gene that produces a toxic protein, resulting in cell death. In this scenario, the promoter produces a toxic protein only under an environmental stimulus specific to the promoter in question. Such a system has been demonstrated by employing a promoter, which becomes active at lower temperatures, ultimately resulting in toxic protein production and cell death.^28^

The envisioned real-world scenario for this assessment would be used as a probiotic in human subjects, which would allow the microbe to be viable and active in the gut at human body temperature (37°C) but not at most ambient temperatures outside the body (i.e., in excreted fecal matter). In this manner, the probiotic can function as designed in the human body (i.e., to produce a therapeutic or to promote gut health), but once outside of the body, the probiotic produces a protein toxic to itself (but not the human host), which kills the engineered cells and thus reduces the chances of uncontrolled growth in sewer systems and other unintended environments.

This scenario was scored moderately for both applicability and feasibility with average assessments of 0.46 and 0.52, respectively (Figure 1). The applicability score benefitted from metrics that reflect a low-cost and limited containment impact on cell growth and function in permissible conditions. The applicability score was ultimately reduced by an undesirable cell escape rate (∼1 escapee per 10^5^ cells).^28^

In contrast, the feasibility was scored highly due to readily available information and build process requirements but scored low for technology readiness and limited availability of test and evaluation (T&E) process requirements. This scenario's assessment fell on the border between the “requires optimization” and “needs basic research development” ranges by agreement of the evaluators.

#### Nutrient dependency (phosphite)

In this scenario, genes encoding enzymes for the uptake of a common necessary nutrient have been removed and replaced with genes that produce an enzyme to metabolize a similar nutrient that is not often found in nature. This scenario was demonstrated in the literature when phosphate (Pi, H_3_PO_4_) transporters were deleted in the cyanobacterium *Synechococcus elongatus* PCC 7942, and phosphite (Pt, H_3_PO_3_) and hypophosphite transporter (HtxBCDE) genes were introduced.

These genes, isolated from other bacterial strains, allowed for the cyanobacterium to grow with Pt as their sole phosphorus source, whereas in turn, knocking out the Pi transporters removed the ability of the cyanobacterium to grow on Pi. Such a system necessitates the addition of Pt to grow, which is not present at appreciable concentrations in nature.^29^ For this scenario, cyanobacteria that can grow in the presence of Pt at an algae farm to produce valuable biochemicals or biofuels^30^ but will not grow outside of that environment was envisioned.

The scenario was scored the highest of any scenario in applicability (0.81) and was scored moderately in feasibility (0.50) as shown in Figure 1. Applicability was scored highly in a number of categories, due in part to the limited cell escape and genetic material escape rates, negligible impact from other organisms on genetic biocontainment, affordable cost of application (∼$1000/metric ton), and lack of toxicity of the nutrient chemical (Pt).^31,32^

The only low score came in species range as phosphite auxotrophy has been limited to cyanobacteria to date. Regarding feasibility, whereas limited development cost and readily available build process requirements resulted in highly scored metrics, information availability and technology readiness were scored low, resulting in a mixed assessment. Ultimately, the evaluators agreed that this genetic biocontainment scenario was on the border between “requires optimization” and “trending to advancement/implementation.”

#### Essential gene (essential yeast)

Genetic biocontainment is achieved in this scenario when essential genes are expressed from a conditional promoter dependent on a chemical added by the end user. A demonstration of this technology was published where two *Saccharomyces cerevisiae* (*S. cerevisiae*) histone H3 and H4 synthetic genes, were placed under transcriptional and recombinational switches triggered by the hormone estradiol.^33^ This scenario presupposes that such a system could be used in an industrial fermentation facility in which estradiol is added to fermentation broth to ensure proper growth for production, but any yeast cells outside of the fermentation broth would fail to survive due to inactivation of the essential genes.

Such a system serves two purposes. First, the risk of the industrial yeast contaminating unwanted ecosystems is reduced through the genetic biocontainment technology embedded. Second, assuming the yeast strain is industrially and economically important (i.e., for producing industrial chemicals at high titer), the intellectual property and trade secrets encoded in the yeast's genome may be harder to recover since it will only grow under certain conditions (i.e., estradiol addition) unknown to a potential unauthorized user. The essential yeast scenario was scored moderately in both applicability and feasibility (0.70 and 0.54, respectively).

Applicability was scored more highly due to a well-controlled cell escape rate (<1 escapee per 10^10^ cells),^33^ affordable cost of chemical inducer ($0.13 per 10,000-L fermenter),^33^ and little containment impact on cell function or growth in permissible conditions. However, the applicability score was adversely affected by containment stability since the publication only reported on containment stability over two rounds of growth.

In addition, estradiol's natural role as a sex hormone and its potential toxic effects to humans and wildlife is a concern.^34^ Feasibility was scored as moderate overall with the availability of build process requirements scoring highly but technology readiness scoring low. There was general agreement that this strategy “requires optimization” but is close to “trending to advancement/implementation.”

#### Nutrient dependency (DAP therapeutic)

A genetic biocontainment scenario similar to the phosphite dependency, where an organism is dependent upon an external supply of a nutrient, was considered as an example of an application close to operational use. In this real-world system that is currently in clinical trials, the dapA gene was deleted from an *E. coli* Nissle strain. This gene is required to generate diaminopimelate (DAP), which is necessary for cell wall biosynthesis and cell growth. With dapA deleted, exogenous DAP is required for the cell to survive.

Such a system has been reduced to practice in Phase II clinical trials in an engineered probiotic treating phenylketonuria (PKU), a condition that results from the inability to degrade phenylalanine, adversely affecting mental and physical development. In this use scenario, the engineered *E. coli* strain is delivered to the gut where DAP is not present. There, the live therapeutic can degrade phenylalanine through genetic modifications made to the organism. The live therapeutic can carry out this role in the gut^8,9^ without reproducing and will eventually die from a lack of DAP supply.

In this manner, the live therapeutic will not be able to colonize effectively in the gut or outside of the body to contaminate sewer systems and broader ecosystems. This scenario was scored highly in both applicability and feasibility (0.71 and 0.60, respectively). Applicability was scored favorably in cell escape rate as the live therapeutic was cleared to nondetectable concentrations in the gastrointestinal tract and in feces of mice within 48 h^9^, and all human participants treated with the live therapeutic in Phase 1/2a clinical trials cleared the bacteria within 4 days^35^ in one of the most rigorous genetic biocontainment studies to date.

The scenario applicability was scored highly due to its limited cost and general safety of the nutrient supplement (in particular because the DAP is not administered with the therapeutic as the treatment does not require engraftment of the probiotic) and containment impact on cell function/growth in permissible conditions. The only categories where applicability was scored low was the ability to measure/monitor containment, which is a complex and laborious task.

The more moderate feasibility score was due to affordable cost and readily available build process requirements being scored highly, whereas the lack of availability of T&E process requirements was scored low. As evidenced with rigorous (but still unfinished) trials, the live therapeutic is on the border of “requires optimization” and “trending to advancement/implementation.”

### Generalized Genetic Biocontainment Categories

Having developed the assessment methodology for genetic biocontainment technologies, generalized categories for genetic biocontainment technologies were created. This process started after collecting >200 publications and patents regarding different genetic biocontainment strategies and grouping them into broad categories. The categorization methodology was based on genetic biocontainment composition. As described earlier, most such technologies have two components: a controller and an actuator of biocontainment.

In this study, the controller regulates activation of the actuator through a stimulus, whereas the actuator implements biocontainment. More specifically, the controller can activate the actuator by regulating its transcription (e.g., as a genetic promoter upstream of a gene encoding the actuator), its translation (e.g., as an aptamer affecting the encoded actuator's ribosome binding site), or its activation (e.g., as a molecular binder sequestering the actuator itself). The actuator is typically a small molecule/nutrient, or a protein generated from a genetic circuit, which either kills or enables survival of the biocontained organism under appropriate conditions.

In this study, the user switch, environmental switch, and no controller were included as controller types. Environmental switches are genetic mechanisms where environmental conditions activate the switch to containment (temperature, pH, light, etc.). User switches are those genetic controllers where the user intervenes to activate the switch to containment (e.g., by adding a chemical inducer). The final type is the case where the containment is intrinsic and requires no genetic switching element (e.g., recoding of an organism such that it requires an nsAA).

In addition, the four predominant actuators, or genetic biocontainment technology categories, considered were essential gene(s), toxin gene(s), nutrient dependency, and genomic recoding. The function of these actuators is described in the specific scenarios earlier. Ultimately, there were six genetic biocontainment categories identified: user switch/essential gene(s), environmental switch/essential gene(s), user switch/toxin gene(s), environmental switch/toxin gene(s), nutritional dependency, and genomic recoding. A summarized description of the containment categories appears in Supplementary Table S7.

The applicability and feasibility assessments for these generalized genetic biocontainment technologies are shown in Figure 2 and Table 2. (Complete assessment scoring can be found in Supplementary Table S2). The highest-scoring candidates for future technological development are nutrient dependency and those encompassing user switches, with an advantage for user switches controlling essential gene biocontainment technologies over user switches actuating toxin gene technologies.

One implication is that environmental switches are inferior to user switches (compare category number 2 with category number 4 in Figure 2). This shortfall is almost exclusively seen in the applicability scores. Another difference observed through this analysis is that essential gene effectors with user switches were generally evaluated as superior to toxin gene effectors with user switches. This difference arises, in part, due to concerns that toxin genes can be deactivated by loss of function mutations.

Finally, although genomic recoding is generally quite applicable, such a system was scored low in feasibility due largely to the difficulty in engineering a genomically recoded organism. Supplementary Table S8 shows the scoring and justification for the genomic recoding example and is the same process as was used in the other general categories as well as the specific scenarios.

### Gap Analysis

The gap analysis indicates areas in which the current genetic biocontainment technologies assessed may not meet requirements. Such potential gaps are determined by the average score of a specific metric across all assessments, including the specific scenarios and generalized categories, and are useful in indicating which aspects of genetic biocontainment may need further development or new technological solutions (Table 3).

**Table 3.** Average normalized feasibility and applicability metric scores across all assessments done in this study

***Feasibility metrics***	***Metric normalized average***
Information availability	0.27
Technology readiness	0.00
Expertise required	0.33
Engineering complexity	0.27
Cost	0.87
Design tool requirements	0.40
Build process requirements	1.00
T&E process requirements	0.20

***Applicability metrics***	***Metric normalized average***
Cell escape rate	0.81
Genetic material escape rate	0.54
Containment stability	0.00
Impact of other organisms	0.76
Cost of application	0.90
Toxicity of application	0.73
Containment impact on cell function	1.00
Ability to monitor containment	0.62
Co-incident	0.24
Species range	0.24

T&E, test and evaluation.

After averaging all of the scores for a metric, those average scores are normalized such that the lowest score is 0, the highest is 1, and all others are decimals between 0 and 1 to allow for a simplified assessment of potential gaps. For the feasibility metrics, the technology readiness has been assessed with the lowest normalized average score of 0, indicating that many of the technologies likely require further development overall before they are ready for real-world applications. The gap analysis of this metric can act as calibration to understand if the field in general still requires development helping to alleviate any concern that the relative scoring of particular technologies in a field is obscuring the fact that it is a nascent or advanced field.

In addition, the testing and evaluation metric has the second lowest assessment in feasibility with a score of 0.2. For the applicability assessments, the containment stability is the lowest scored metric with a normalized average score of 0. This is largely due to the fact that most studies have not tested containment stability over many generations. In addition, the species-range metric was scored low at 0.24, indicating that many genetic biocontainment technologies are not expected to be portable across a multitude of organisms.

## Discussion

An assessment methodology and standardized metrics can be useful for evaluating the prospective technologies in a particular field during the development stage, allowing for the prioritization of diverse emerging technologies. Such metrics will be enabling to the biosafety and biosecurity communities, who will increasingly be called upon to evaluate genetic biocontainment strategies for engineered microbes in a diverse array of environments.

For this study, scenarios were developed to provide specific examples of the general methods of genetic biocontainment. The scenarios that were created helped test the assessment methodology, including the specific metrics. The framework was then applied to evaluate generalized genetic biocontainment strategies. Expert opinion and a metric-based scoring system were used resulting in a semiquantitative comparative assessment.

Although this method does rely on SMEs there is precedent for this type of analysis in biological research.^17^ The relative scores of disparate technologies produced from this method allowed for the prioritization of those genetic biocontainment technologies that can aid in deciding how to allocate resources for future development based on application requirements. In addition, the gap analysis provided insight into possible operational requirements that are not met with the current genetic biocontainment technologies.

Information that can influence the future resource allocation for genetic biocontainment methods can be seen in Figure 2. For example, based on the category assessments, one would likely choose to continue development of essential gene-based biocontainment over toxin-mediated containment for the release of an engineered organism. The lower priority for toxin-based biocontainment is due in part to the possible (and in some cases likely) failure mode for such systems where the toxin gene or its controller becomes mutated, rendering them ineffective.

A synthetic toxin gene and its activation serves little purpose to the engineered organism, which is evolutionarily programmed to persist and grow, such that the growth disadvantage from the toxic gene product causes the cell to mutate the toxin gene or the controller element DNA.^36^ However, in the scenario of essential gene-based biocontainment, an essential gene is necessary for the organism to grow and thrive, and, therefore, it is evolutionarily advantageous to the organism to retain the essential gene.

Whereas the containment system can experience a failure mode in which the essential gene is constitutively expressed rather than conditionally expressed, switch design can be implemented in which many genetic changes must occur to cause such a failure.

As with the biocontainment actuator, the assessment and prioritization can also indicate the use of particular genetic controller elements for continued development over others. As indicated in Figure 2, both essential genes and toxin genes with user-controlled switches were scored highly when compared with those with environmental switches. One contributing factor to this assessment is that environmental conditions are subject to change in ways that are difficult to anticipate, particularly in natural settings.

For instance, a controller (inducible promoter) triggered by temperature may act in an unexpected manner due to high variances in natural environments, such as those seen in recent years due to climate change. In contrast, user switches enable greater control over the genetic biocontainment system and are expected to be more predictable.

An interesting approach to prioritization is to focus on those technologies that may be promising in applicability but limited in feasibility. This allows for the further development of the features that contribute to feasibility to bring to fruition a promising technology. As can be seen in Figures 1 and 2, genomic recoding is one such technology category that may enable strong genetic biocontainment but needs development to facilitate feasibility in implementation.

Engineering a cell to remove a codon from the entirety of the genome, freeing that codon for use with an orthogonal translational system, requires significant effort.^3^ Similarly, encoding nsAAs within an essential gene such that the gene product still functions but is dependent on the presence of nsAAs, requires protein engineering expertise and execution.^37^ Considering such efforts, portability of this technology to many different, potentially less genetically tractable organisms, is difficult.

Although the applicability is generally high for this technology, how successfully it can be deployed will be dependent on the application. In a large environmental application, for example, the safety, environmental impact, and cost of the unnatural nutrients used can hamper applicability, as in the case of many nsAAs. Developing methods to mitigate such feasibility shortcomings could thus enable a potentially powerful biocontainment technology.

The assessment methodology also indicates gaps that can be targeted for further technical development. One particular gap in the development of the genetic biocontainment technologies assessed in this study was indicated by the low relative average scores for the species range metric in the gap analysis. By choosing this metric in the first place, the assertion is being made that there is value in developing genetic biocontainment technologies that ease portability across organisms (for some users of the framework, this factor may be less important; such users can choose to prioritize a single organism of choice by excluding that metric).

The assessment methodology highlighted that very few reviewed studies focused on developing genetic biocontainment technologies that are easily portable from one organism to another. An interesting instance of a portable containment mechanism that also addresses the feasibility of genomic recoding is a recoding scheme that creates the requirement for nsAAs in diverse bacteria without removing a codon from the entirety of the genome.^22^

Another shortcoming that was elucidated from the gap analysis of the scenarios and categories assessed is containment stability. This metric represents how long the containment can last when deployed in its intended operational environment. Genetic biocontainment can be broken by a number of conditions such as cross-feeding (where another organism supplies needed nutrients), genetic mutations, or unexpected environmental conditions, all of which must be considered in order for containment to be consistent and stable.

Most studies do not measure the escape rate beyond several days and, therefore, it is difficult to know how stable the genetic biocontainment technologies are. Experiments targeting the long-term stability of the containment are needed both in laboratory settings (such as in long-term batch culture) as well as in testbeds that simulate the conditions of the environment in which the engineered organism would be released (e.g., a mesocosm of a particular environment). Such testbeds are also indicated as a need in the gap analysis as demonstrated by the low average score for testing and evaluation requirements.

## Conclusions

It is the intention that this framework can be used in a variety of ways. First, it can be used directly as is for the evaluation of genetic biocontainment approaches, applying the feasibility and applicability metrics to a proposed solution. Although an individual may use the framework on their own, it is recommended that the user employ at least a small group of SMEs to sample a greater range of opinion. The team approach can help identify where there is general agreement and where there is greatest disagreement. It can also spur productive discussion, which further highlights the strengths and shortcomings of a given proposed solution.

Second, the framework can be adapted to the particular needs of a given situation by (1) adding weights to some metrics deemed particularly important; (2) adjusting the parameters of the bins used to describe each metric (e.g., cost requirements may vary widely); and (3) removing some metrics entirely and/or adding new ones. Third, the format of this framework can be applied to distinctly different challenges in biotechnology prioritization, where some of the metrics employed in this study will likely remain relevant, but new metrics are also likely to be needed.

The development of an assessment methodology with defined metrics provided us with a straightforward system for evaluating genetic biocontainment strategies intended for various real-world applications and with insights into existing gaps in genetic biocontainment strategies. The literature review carried out for this assessment indicates that such assessments are conspicuously absent in the field of biotechnology, even though they are widespread in the development of mechanical and electrical systems, particularly in aerospace applications^38,39^ and in U.S. military applications.^40^

As biotechnology continues to mature from a discipline often reliant on trial and error to a predictable engineering discipline akin to mechanical or electrical engineering, the utility of such assessments increases. This type of methodology can be particularly useful when applied to biological systems, as the diversity of biology allows for many different technological solutions to be applied to address the same challenges. Prioritization can be invaluable in determining where to apply resources to develop the nascent technologies that show the most promise.

## Glossary

Genetic biocontainment technologies—Technologies utilizing embedded genetic methods to implement biocontainment such that an organism is restricted to living and growing only under a specified set of conditions.

Genetic biocontainment scenario—An example in which a particular genetic biocontainment technology is utilized.

Genetic biocontainment categories—Categories into which genetic biocontainment technologies are grouped according to shared features in common, such as conditional dependence on an essential gene.

## Supplementary Material

Supplementary Figure S1

Supplementary Table S1

Supplementary Table S2

Supplementary Table S3

Supplementary Table S4

Supplementary Table S5

Supplementary Table S6

Supplementary Table S7

Supplementary Table S8

**Supplemenat Material is available at:** https://www.liebertpub.com/doi/10.1089/apb.2023.0025.0025

## Distribution Statement

Approved for public release. Distribution is unlimited. © 2023 Massachusetts Institute of Technology. Delivered to the U.S. Government with Unlimited Rights, as defined in DFARS Part 252.227-7013 or 7014 (Feb 2014). Notwithstanding any copyright notice, U.S. Government rights in this study are defined by DFARS 252.227-7013 or DFARS 252.227-7014 as detailed above. Use of this study other than as specifically authorized by the U.S. Government may violate any copyrights that exist in this study.
