# Supplementary figures and images for: A Methodology for the Assessment and Prioritization of Genetic Biocontainment Technologies for Engineered Microbes
Source: Appl Biosaf. 2024 Jun 20;29(2):108–19. doi: 10.1089/apb.2023.0025 (PMC11319856; doi:10.1089/apb.2023.0025)

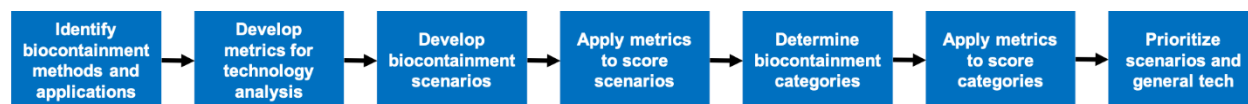

*Figure S1. Process steps for the assessment of genetic biocontainment technologies.*

Supplement: Supplementary Figure S1 [file apb.2023.0025_suppl_figs1.pdf]
