# Supplementary material for: A Methodology for the Assessment and Prioritization of Genetic Biocontainment Technologies for Engineered Microbes
Source: Appl Biosaf. 2024 Jun 20;29(2):108–19. doi: 10.1089/apb.2023.0025 (PMC11319856; doi:10.1089/apb.2023.0025)
Supplement: Supplementary Table S1 [file apb.2023.0025_suppl_tables1.pdf]

	**Synthetic Organisms**	**Biocontainment**	**Optional Terms** (used occasionally, but led to many irrelevant results)
**Keywords** (used for all databases)	(bacteria OR virus OR *organism OR microb*) W/2 (synthetic OR *engineered OR *designed OR recoded OR re-coded OR "de novo" OR alien OR xeno* OR unnatural OR "not natural" OR noncanonical OR non-canonical OR "new to nature")	• *contain<br>• *containment<br>• *firewall<br>• *safety<br>• safeguard<br>• "kill switch"<br>• deadman	• suicid*<br>• protect*<br>• restric*<br>• restrain*<br>• constrain*
**Medical Subject Headings** (mesh terms) (used for PubMed)	• "Bacteria/genetics"[mesh]<br>• "Bioengineering"[mesh]<br>• "Biotechnology"[mesh]<br>• "Genetic engineering"[mesh]<br>• "Metabolic Engineering"[mesh]<br>• "Organisms, Genetically Modified"[mesh]<br>• "Synthetic Biology"[mesh]	• "Biohazard Release/prevention & control"[mesh]<br>• "Containment of Biohazards/methods"[mesh]<br>• "Genes, Lethal"[mesh]<br>• "Safety"[mesh]<br>• "Microbial Viability/genetics"[mesh]	• "Xenobiotics"[mesh]

Table S1. Search terms used to create the bibliography of references to determine the metrics, scenarios, categories and specifics of the genetic biocontainment technologies assessed
