# Supplementary material for: A Methodology for the Assessment and Prioritization of Genetic Biocontainment Technologies for Engineered Microbes
Source: Appl Biosaf. 2024 Jun 20;29(2):108–19. doi: 10.1089/apb.2023.0025 (PMC11319856; doi:10.1089/apb.2023.0025)
Supplement: Supplementary Table S2 [file apb.2023.0025_suppl_tables2.zip › Suppl_TableS2.docx]

*Table S2 (Separate File). List of publications, patents, and patent applications relevant to genetic biocontainment. These references were reviewed by the authors and informed their analysis regarding state-of-the-art genetic biocontainment technologies and their categorization.*
