# Supplementary material for: A Methodology for the Assessment and Prioritization of Genetic Biocontainment Technologies for Engineered Microbes
Source: Appl Biosaf. 2024 Jun 20;29(2):108–19. doi: 10.1089/apb.2023.0025 (PMC11319856; doi:10.1089/apb.2023.0025)
Supplement: Supplementary Table S3 [file apb.2023.0025_suppl_tables3.pdf]

**Release Conditions**	**Containment Technology**	**Controller**	**Containment Details**
Human body	Nutrient Dependency	User Induced	Technology Readiness Level
Open Environment	Essential Gene	Environmentally Induced	Escape Rate
Semi-Open Environment	Toxin Gene	None	Organism Portability
Closed Environment	Genomic Recoding	-	-

Table S3. Information considerations for scenario development. Each scenario used information from each of four categories (Release Condition, Containment Technology, Controller, and Containment Details) to reduce overlap and produce unique scenarios.
