# Supplementary material for: A Methodology for the Assessment and Prioritization of Genetic Biocontainment Technologies for Engineered Microbes
Source: Appl Biosaf. 2024 Jun 20;29(2):108–19. doi: 10.1089/apb.2023.0025 (PMC11319856; doi:10.1089/apb.2023.0025)
Supplement: Supplementary Table S4 [file apb.2023.0025_suppl_tables4.pdf]

	1	2	3
**Information Availability**	Limited (e.g., Few publications, database entries)	Moderate (e.g., Moderate publications, database entries)	Wide (e.g., Many publications, database entries)
**Technology Readiness**	Research and Development (e.g., DoD RDT&E Budget Category 6.1 – 6.3)	Demo & Validation; Engineering development (e.g., DoD RDT&E Budget Category 6.4 and above)	Operational systems Available
**Expertise Required**	Expert (e.g., Requires specific experience with particular technology)	Intermediate (Synthetic biology experience)	No Specific Training (General biology lab expertise)
**Engineering Complexity**	High (Newly engineered system)	Medium (Minor modification of available system)	Low (little or no engineering efforts)
**Cost**	High (E.g. >$10,000,000)	Moderate (E.g. ~$1,000,000)	Low (E.g. <$1,000,000)
**Design Tool Requirements**	Custom design tool required	Existing design tool required	Little to no design tool requirement
**Build Process Requirements**	Requires custom equipment and materials (Not commercially available)	Existing but specialized equipment and materials (Expensive, limited availability)	Common equipment and materials (Affordable, easy access)
**T&E Process Requirements**	Custom T&E required (New bioinformatic/screening methods, specialized equipment)	Existing T&E required (e.g., Automated screening and bioinfo pipeline, common equipment)	Little to no T&E required

[red] = Hinders containment **[yellow] = Equally weighted** **[green] = Enables containment**

Table S4. Feasibility metrics for determining the ease of engineering for a genetic biocontainment technology. This is based on the design, build, test and learn cycle. Brief descriptions of the interpretations of the scoring are included in the columns under each score.
