# Supplementary material for: A Methodology for the Assessment and Prioritization of Genetic Biocontainment Technologies for Engineered Microbes
Source: Appl Biosaf. 2024 Jun 20;29(2):108–19. doi: 10.1089/apb.2023.0025 (PMC11319856; doi:10.1089/apb.2023.0025)
Supplement: Supplementary Table S5 [file apb.2023.0025_suppl_tables5.pdf]

	1	2	3
**Cell escape rate**	> 1 in 10^8	At or near 10^8	< 1 in 10^8
**Genetic material escape rate**	Likely	Possible	Unlikely
**Containment Stability**	Hours - Days	Weeks - Months	Years - Permanent
**Impact of other organisms**	Likely to allow for break of containment	Possible but unlikely to allow break of containment	No affect or helps containment
**Cost of Application (e.g. inducers)**	Expensive	Moderately expensive	inexpensive
**Toxicity of Application (e.g. inducers, byproducts)**	Toxic to humans or environment	Toxic to some environments	Non-toxic
**Containment impact on cell function/growth**	High impact (i.e. cell function or grow severely limited)	Moderate impact (i.e. limited cell function or growth)	Low impact (i.e. minimal affect on cell function or growth)
**Ability to measure/monitor containment**	Difficult (no current method to measure escape or duration)	Moderate (measure escape or duration but not both)	Easy
**Co-incident**	Needs to be maintained by bystander (e.g. add inducer)	Triggered automatically at the time of need (condition dependent switch)	Containment is inherent and continuous
**Species Range**	Works in one organism	Works in a small set of organisms	Works in many organisms

Table S5. Applicability metrics for determining the ease of use and effectiveness in an application. Brief descriptions of the interpretations of the scoring are included in the columns under each score.
