# Supplementary material for: A Methodology for the Assessment and Prioritization of Genetic Biocontainment Technologies for Engineered Microbes
Source: Appl Biosaf. 2024 Jun 20;29(2):108–19. doi: 10.1089/apb.2023.0025 (PMC11319856; doi:10.1089/apb.2023.0025)
Supplement: Supplementary Table S6 [file apb.2023.0025_suppl_tables6.pdf]

	Eval 1 cryodeath	Eval 1 gencode	Eval 1 DAP	Eval 1 phosphite	Eval 1 Essential Yeast	Eval 2 cryodeath	Eval 2 gencode	Eval 2 DAP	Eval 2 phosphite	Eval 2 Essential yeast	Eval 3 cryodeath	Eval 3 gencode	Eval 3 phosphite	Eval 3 essential yeast	Eval 3 DAP	Essential genes env/user	Nutrient synthetic	Toxin env/ user	Nutrient	Normalized Average
**Information Availability**	3	1	2	1	2	3	1	2	1	2	2	1	1	2	2	1	2	2	3	0.27
**Technology Readiness**	1	1	2	1	1	1	1	2	1	1	1	1	2	1	2	1	1	2	3	0.00
**Expertise Required**	2	1	2	2	2	2	1	2	2	2	2	1	2	2	3	2	1	2	3	0.33
**Engineering Complexity**	2	1	2	2	2	2	1	2	2	2	2	1	1	2	2	2	1	2	3	0.27
**Cost**	3	2	3	3	3	3	2	3	3	2	3	2	3	3	3	3	2	3	3	0.87
**Design Tool Requirements**	2	2	2	2	2	2	2	2	2	2	2	2	2	2	2	2	1	2	3	0.40
**Build Process Requirements**	2	3	3	3	3	3	3	3	3	3	3	3	3	3	3	3	3	3	3	1.00
**T&E Process Requirements**	1	2	1	2	2	1	2	1	2	2	1	1	2	2	2	2	2	2	2	0.20

	Eval 1 cryodeath	Eval 1 gencode	Eval 1 DAP	Eval 1 phosphite	Essentil Yeast	Eval 2 cryodeath	Eval 2 gencode	Eval 2 DAP	Eval 2 phosphite	Essential yeast	Eval 3 cryodeath	Eval 3 gencode	Eval 3 phosphite	essential yeast	Eval 3 DAP	Essential genes user	Essential genes Env	Nutrient synthetic	Toxin env	Toxin user	Nutrient	Normalized Average
**Cell escape rate**	1	3	3	3	3	1	3	3	3	3	1	3	3	3	3	3	1	3	1	2	2	0.81
**Genetic material escape rate**	2	3	2		2	1	3	1	3	NA	2	3	2	2	2	2	2	3	2	2	2	0.54
**Containment Stability**	2	2	2	2	1	2	2	1	2	1	2	2	2	1	1	1	1	2	1	1	3	0.00
**Impact of other organisms**	2	3	2	3	2	1	3	2	3	2	3	3	3	3	2	2	2	2	3	3	1	0.76
**Cost of Application**	3	1	3	2	3	3	1	3	3	3	3	1	3	3	3	3	3	1	3	3	2	0.90
**Toxicity of Application**	3	1	3	3	3	3	1	3	3	1	3	1	2	1	3	3	NA		3	2	2	3	0.73
**Containment impact on cell function/growth**	2	2	3	1	3	3	2	3	2	3	3	3	3	3	3	3	3	2	3	2	3	1.00
**Ability to measure/monitor containment**	2	2	1	2	3	2	2	1	2	2	2	2	2	2	2	3	3	3	3	3	3	0.62
**Co-incident**	2	2	2	1	1	3	2	2	2	2	2	2	3	2	2	1	2	2	2	1	1	0.24
**Species Range**	2	1	2	2	2	2	2	1	1	2	2	2	1	2	2	3	2	2	2	1	3	0.24
