# Supplementary material for: A Methodology for the Assessment and Prioritization of Genetic Biocontainment Technologies for Engineered Microbes
Source: Appl Biosaf. 2024 Jun 20;29(2):108–19. doi: 10.1089/apb.2023.0025 (PMC11319856; doi:10.1089/apb.2023.0025)
Supplement: Supplementary Table S7 [file apb.2023.0025_suppl_tables7.pdf]

| | General Category | | | |
|---|---|---|---|---|
| | **Genomic Recoding** | **Essential Gene** | **Toxin Gene** | **Nutrient** |
| **Biocontainment Technology Description**<br><br>**(Actuators)** | One codon and its translation machinery removed from genome<br><br>Orthogonal translation loads nsAA into essential protein at free codon<br><br>Cell requires nsAA to survive | Essential gene is expressed by a genetic switch<br><br>Cell only grows when switch is ON in permissive conditions | Toxin gene is expressed by a genetic switch<br><br>Cell only grows when switch is OFF in permissive conditions | Gene whose product helps make an essential molecule is knocked out<br><br>Cell only grows when essential nutrient is supplied to the cell |
| **Controller Options** | No genetic switch controller necessary | Environmental condition or user induced genetic switch | Environmental condition or user induced genetic switch | No genetic switch controller necessary |

*Table S7. A summarized description of the generalized categories of genetic biocontainment, including the genetic biocontainment technology description and the controller for each category.*
