# Supplementary material for: A Methodology for the Assessment and Prioritization of Genetic Biocontainment Technologies for Engineered Microbes
Source: Appl Biosaf. 2024 Jun 20;29(2):108–19. doi: 10.1089/apb.2023.0025 (PMC11319856; doi:10.1089/apb.2023.0025)
Supplement: Supplementary Table S8 [file apb.2023.0025_suppl_tables8.pdf]

Metric	1	2	3	Justification
Information Availability	Limited<br><br>(e.g., Few publications, database entries)	Moderate<br><br>(e.g., Moderate publications, database entries)	Wide<br><br>(e.g., Many publications, database entries)	"Genomically recoded organisms"=101 results<br><br>"Non-standard amino acid"=139 results<br><br>Pubmed search 10/18/23
Technology Readiness	Research and Development<br><br>(e.g., DoD RDT&E Budget Category 6.1 – 6.3)	Demo & Validation; Engineering development<br><br>(e.g., DoD RDT&E Budget Category 6.4 and above)	Operational systems Available	Mostly in academic literature<br><br>The companies GRO Biosciences and Pearl Bio use genomic recoding.
Expertise Required	Expert<br><br>(e.g., Requires specific experience with particular technology)	Intermediate<br><br>(Synthetic biology experience)	No Specific Training<br><br>(General biology lab expertise)	Recoding is arduous, has a number of unintended consequences, and requires lots of tuning and sensitivity analysis. [1]
Engineering Complexity	High<br><br>(Newly engineered system)	Medium<br><br>(Minor modification of available system)	Low<br><br>(little or no engineering efforts)	See above
Cost	High<br><br>(E.g. >$10,000,000)	Moderate<br><br>(E.g. ~$1,000,000)	Low<br><br>(E.g. <$1,000,000)	To start a recoding project from scratch (as needed for plant symbionts) would require several million dollars. Sc2.0 is already over $3 million [2, 3]
Design Tool Requirements	Custom design tool required	Existing design tool required	Little to no design tool requirement	Some recoding tools exist. [4]
Build Process Requirements	Requires custom equipment and materials<br><br>(Not commercially available)	Existing but specialized equipment and materials<br><br>(Expensive, limited availability)	Common equipment and materials<br><br>(Affordable, easy access)	Nothing unusual required for build processes including DNA synthesis, Restriction enzyme based assembly. [5]
T&E Process<br><br>Requirements	Custom T&E required (New bioinformatic/screening methods, specialized equipment)	Existing T&E required (e.g., Automated screening and bioinfo pipeline, common equipment)	Little to no T&E required	Would require field testing to ensure it is working as designed.

Table S8. Feasibility metrics for determining the ease of engineering for a genetic biocontainment technology. An example of the scoring from an SME and the justification of the scores is shown in the last column.

References

1. Fredens, J., et al., *Total synthesis of Escherichia coli with a recoded genome.* Nature, 2019. **569**(7757): p. 514-518.
2. *Complete synthesis of designer eukaryotic genome, Sc2.0*. 2022 [cited 2023 11/03/2023]; Available from: https://www.nsf.gov/awardsearch/showAward?AWD_ID=1616111.
3. Mullin, E. *Designer Chromosomes Point to New Synthetic Life-Forms*. MIT Technology Review, 2017.
4. Schwartz, A.C.D., D.; Isaacs, F.; Stracquadanio, G. *This CAD program can design new organisms*. IEEE Spectrum, 2021.
5. Lajoie, M.J., et al., *Genomically recoded organisms expand biological functions.* Science, 2013. **342**(6156): p. 357-60.
